# Case study on synergistic development strategy of cross-border e-commerce and logistics: An empirically model estimation

**DOI:** 10.1371/journal.pone.0304393

**Published:** 2024-06-11

**Authors:** Xiaolie Qi, Wen Qin, Baojun Lin

**Affiliations:** 1 South China Business College Guangdong University of Foreign Studies, Guangzhou, China; 2 The Hong Kong Polytechnic University, Hong Kong, China; 3 Guangdong Polytechnic of Science and Technology, Zhuhai, China; 4 School of Business, City University of Macau, Macau, China; 5 OSEA Alliance Group, Brisbane, Australia; Sichuan Agricultural University, CHINA

## Abstract

This study investigates the dynamic relationship between cross-border e-commerce and logistics co-development strategies within the context of small and medium-sized enterprises (SMEs) in China. The primary objective is to formulate an empirical model capable of estimating the utility and flexibility of cross-border e-commerce logistics, specifically focusing on its role in achieving competitive advantage for SMEs. The research employs a comprehensive approach, considering various factors that influence the formulation and implementation of cross-border e-commerce logistics strategies. Factors such as scale, quality, potential, and infrastructure are scrutinized to provide a nuanced understanding of the dynamic interplay. Real-world data is analyzed using advanced statistical techniques to derive meaningful insights. The data of CBEC and logistics planning in Guangdong from 2013 to 2018 was used as a case example to demonstrate its and flexibility and usefulness of empirically proposed model estimation and develop a holistic indicator system for the evaluation of the synergistic development of CBEC and logistics. This research focus on the modelling of the CBEC for the synergistic effect analysis and evaluate the usefulness and flexibility of CBEC to achieve competitive advantage. The study aims to uncover insights into the most effective approaches for Chinese SMEs in navigating the landscape of cross-border e-commerce. By examining the results derived from the empirical model, the research sheds light on the impact of cross-border e-commerce logistics on competitive advantage. The findings contribute to a deeper understanding of how SMEs can strategically position themselves in the cross-border e-commerce arena. Through the analysis of real-world data and the application of advanced statistical methods, this research offers valuable insights for Chinese SMEs. The insights generated from the study not only illuminate the intricacies of cross-border e-commerce logistics but also provide practical recommendations to reduce associated costs. Ultimately, the study aims to equip SMEs with the knowledge necessary to thrive in the evolving landscape of cross-border e-commerce.

## Introduction

Cross-border electronic commerce (also known as CBEC) is becoming more and more common, and cross-border electronic logistics is being carefully monitored for integration process. One of the most significant advantages of CBEC is its ability to transcend the geographical boundaries of traditional commerce. By 2020, more than 2 billion e-shoppers, or 60% of the targeted worldwide population, would have transacted 13.5% of their total retail consumption online, equating to a market value of US$3.4 trillion. In China, the growth of internet technology has created opportunities for the development of e-commerce. Since Chinese governmental authority boards reviewed its CBEC legislation in 2014, CBEC transactions have generated huge profits [1]. In addition, the political and economic background of the trade war between the United States and China imposed an enormous amount of pressure on CBEC for small and medium-sized enterprises (SMEs) [2]. To deal with the complexity of the supply chain, SMEs must now develop a plan to handle their direct and indirect customers with accessible, pleasant, and personalised logistics services [3].

For instance, in 2018, the yearly transaction value of CBEC in China grew by 8.5% year-over-year to 31.63 trillion yuan, representing an outstanding result [3]. The generated value of CBEC imports and exports has surged by over 50% annually [2]. China’s international trade and economic growth are transformed and accelerated by the CBEC market [4]. Specifically, the development of foreign trade has a very long history in one of the provinces in China, i.e., Guangdong. The Chinese province has the enormous geographical advantage of facing Southeast Asia, the support of the Pearl River Delta in its hinterland, and proximity to Hong Kong and Macau [5]. Therefore, robust manufacturing sectors have been established in the province to facilitate CBEC [6]. Those foreign trade SMEs are increasingly engaged in international and domestic e-commerce. More than 200,000 domestic enterprises engage in cross-border businesses via e-platforms. Approximately 70% of the nation’s CBEC businesses are situated in Guangdong, with SMEs and sole proprietorships accounting for approximately 50% [7]. 50% of CBEC enterprises are SMEs and individual operators; they have opened over 300,000 online store platforms in major CBEC, of which 80% have completed full online marketing [5]. This has accounted for substantial import and export markets for approximately 30% of the country’s total imports and exports, with a relatively complete industrial chain [6]. Various production bases in the Pearl River Delta cover the complete supply chain of almost all the best-selling products in Greater China, including those commercial products of apparel, footwear, food, tobacco and alcohol, and daily chemicals, in addition to diverse culture, transportation, etc. [5]. The sales domain for international e-commerce is diverse and complex [8]. The importance and objective of developing an empirical model that estimates the utility and flexibility of cross-border e-commerce logistics in achieving competitive advantage in China.

Cross-border logistics often involves dealing with regulatory, customs, and geopolitical complexities [9]. An empirical model can help assess potential risks and uncertainties, enabling businesses to devise contingency plans and minimize disruptions to their operations. Due to the complexity of CBEC markets, logistics planning is more complex than traditional e-commerce, which impacts the customer experience for order fulfilment and delivery [10–12]. Guangdong’s cross-border e-commerce imports and exports accounted for 44.19 billion RMB in 2017, ranking first in scale [13]. Comprehensive pilot zones for CBEC markets were established in four Guangdong cities, namely Shenzhen, Guangzhou, Zhuhai, and Dongguan [14]. More synergy is required for the CBEC markets with supply and demand planning in Guangdong at this time [14]. The rapid expansion of the CBEC market can then boost the requirements for cross-border logistics in terms of cost and speed. The relatively slow growth of cross-border logistics may be hindered the expansion of CBEC [8].

Moreover, China has established several pilot zones to encourage cross-border e-commerce [8]. These zones enjoy preferential policies, facilitating smoother customs clearance and fostering international trade. The government has implemented favourable tariff and tax policies for cross-border e-commerce transactions, reducing costs for consumers and promoting the growth of the sector [9]. The Chinese government has demonstrated a commitment to policy stability in the cross-border e-commerce space, providing a conducive environment for businesses to thrive [8]. China’s cross-border e-commerce market has experienced exponential growth, driven by increasing consumer demand for international products [3]. There is a noticeable shift in consumer behaviour towards online shopping, with a preference for foreign goods [11]. This trend is supported by a rising middle class with increasing disposable income. Social media platforms and e-commerce are increasingly integrated, creating new avenues for cross-border transactions [9]. The widespread adoption of digital payment systems like Alipay and WeChat Pay has facilitated seamless cross-border transactions. The use of big data and analytics has improved supply chain management, inventory control, and personalized marketing strategies in the cross-border e-commerce sector [15]. China has invested heavily in building state-of-the-art logistics and fulfillment centres, optimizing the storage and distribution of cross-border e-commerce goods. The development of efficient transportation networks, including high-speed railways and modern airports, has facilitated the timely movement of goods across borders [14]. Adhering to diverse international regulations remains a challenge for businesses engaged in cross-border e-commerce. The market is highly competitive, driving companies to innovate in areas such as supply chain optimization, last-mile delivery, and customer experience [16]. Collaborations and partnerships with global e-commerce platforms and logistics providers present opportunities for expansion. The cross-border e-commerce and logistics sectors in China are expected to continue their robust growth, driven by technological advancements and evolving consumer preferences [8]. China’s cross-border e-commerce ecosystems and logistics capabilities have evolved rapidly, fuelled by supportive economic policies, changing consumer behaviour, technological innovations, and substantial infrastructural developments. While challenges exist, the overall landscape presents immense opportunities for businesses and investors in the dynamic and growing market [15].

The landscape of Cross-Border E-commerce (CBEC) and its intricate connection with logistics presents a complex terrain that, to our knowledge, has seen limited empirical model estimation. This is particularly pertinent given the nascent stage of CBEC in many countries, where its synergistic development remains at a notably low level, impeding the seamless operation and growth of cross-border logistics [7].

A crucial case in point is the Chinese province of Guangdong, standing at the crossroads of international e-commerce evolution in the coming decades. Recognizing the imperative for improved synergy between international e-commerce and logistics, the province faces a pivotal moment. This study, therefore, seeks to address the overarching research question: How can the proposed empirical model estimation contribute to assessing the synergetic performance of CBEC and logistics?

To unravel this question, we leverage panel data spanning from 2013 to 2018, focusing on CBEC and logistics planning in Guangdong. This empirical exploration serves as a compelling case example, showcasing the flexibility and utility of the proposed model estimation. Our aim is to forge a holistic indicator system that evaluates the synergistic development of CBEC and logistics, shedding light on the intricacies of their interplay.

Beyond a mere assessment, this study delves into understanding the factors that exert influence on the synergistic development within the Chinese province. By offering effective and practical insights into CBEC, we contribute to the body of knowledge, especially relevant for foreign trade enterprises seeking to capitalize on the policy dividends of CBEC development. The overarching goal is to transform conventional trade paradigms and advance global economic integration.

The empirically proposed model estimation for synergistic development assumes a pivotal role in providing theoretical support and rationale for decision-making. Specifically, it caters to the government authorities responsible for formulating policies. These policies, informed by a nuanced understanding of the synergies between CBEC and logistics, aim to foster a combined and mutually beneficial growth trajectory. In essence, this research not only scrutinizes the current landscape but also lays the foundation for strategic decision-making to propel the symbiotic evolution of CBEC and logistics.

The remaining part of this paper is organised as follow. First, the case studies on the synergistic development of CBEC are presented and then followed by the reviews of related research of e-commerce platforms and cross-border logistics. Second, the evaluation of the synergistic effects of CBEC and empirical model estimation are presented. On this basis, a scientific and reasonable evaluation index system for the synergistic development of CBEC is constructed with the support of literature and quantitative analyses. Finally, the study evaluates and analyses the efficiency of the synergistic effect of CBEC by use of a case study from one of the Chinese provinces, i.e., Guangdong and determine the synergistic mechanisms of CBEC and logistical activities. A concluding remark for effectiveness of synergistic development of CBEC by use of proposed model estimation is made.

## Research lenses

### Cross-border e-commerce

Cross-border e-commerce (CBEC) consists mainly of buyers, sellers, cross-border online platforms and cross-border logistics providers and payment providers and consists of cross-border logistics transactions conducted by multiple parties from different tariff zones through e-commerce platforms [15]. CBEC has considerably evolved to promote the economic growth of several nations in the globe. As is increasingly observed, to increase the efficiency of logistics service in CBEC and hence minimise the costs associated with this process, SMEs opt to conduct an intensive review of their logistics service and performance to improve their competitiveness. However, the proportion of published literature on e-commerce and logistics performance remains low. Much of the existing literature on CBEC focuses on applied research and empirical model estimation to support practical applications. These studies aim to analyze the dynamics of CBEC transactions, logistics operations, and their interplay with various factors such as market conditions, regulatory frameworks, and technological advancements [16, 17]. Moreover, the birth of cross-border e-commerce has shortened the traditional general trade transaction chain, reduced the transaction cost of the whole chain and enhanced the transaction efficiency [18]. Along with the gradual improvement of cross-border e-commerce infrastructure, the development of cross-border e-commerce has gradually accelerated, with a growth rate that exceeds that of traditional foreign trade and continues to give rise to more efficient mode innovation [19]. The evolution of CBEC has led to the emergence of different modes of logistics fulfillment chains to meet the diverse needs of goods transportation. Traditional international freight forwarding modes are gradually being supplemented or replaced by innovative models such as overseas warehousing and direct mailing [18, 19]. Around the evolution of trade forms and categories, the market has developed to form different modes of logistics fulfilment chains to meet the logistics needs of different goods. Along with the rapid development of cross-border e-commerce, the original international freight forwarding mode that serves traditional traders cannot fully meet the demand, and new modes such as overseas warehousing and direct mailing are growing [20]. The accelerated globalization of China’s domestic manufacturers or brands necessitates deeper integration of domestic logistics enterprises into overseas markets. This trend not only requires collaboration with international logistics partners but also entails the restructuring of logistics resource systems to meet the evolving demands of cross-border trade [21]. The COVID-19 pandemic has significantly impacted global consumption habits, leading to an increased penetration rate of cross-border e-commerce businesses. This trend underscores the importance of providing comprehensive international logistics services, including procurement, inventory management, and warehousing, to meet the growing demand [22, 23]. In addition to international express delivery and some special lines, most cross-border e-commerce logistics companies need to collaborate with multiple third-party logistics providers in order to complete the full chain of delivery.

In the realm of global trade, the digital age has spawned a transformative phenomenon—Cross-Border E-commerce (CBEC). At the forefront of this revolution stands China for its remarkable strides in technology and commerce. China’s journey into Cross-Border E-commerce can be traced back to the early 2000s when the global proliferation of the internet laid the foundation for a new era of international trade [24]. Technological innovation plays a pivotal role in shaping the trajectory of CBEC expansion. China, in particular, has leveraged cutting-edge technologies such as artificial intelligence, blockchain, and fintech to enhance logistics efficiency, ensure supply chain transparency, and drive the integration of e-commerce platforms [24–26]. Rapid economic growth, supportive government policies, and a tech-savvy population propelled CBEC’s evolution from a mere experiment to a formidable economic force [17]. The impact of CBEC on China’s economy has been profound. It facilitated the exposure of domestic brands to an international audience, enabling them to transcend geographical boundaries [25]. This, in turn, fuelled economic growth, created employment opportunities, and spurred technological innovation. While CBEC presents significant opportunities for economic growth and international trade, it also poses challenges related to intellectual property protection, regulatory complexities, and interoperability issues. Addressing these challenges requires thoughtful solutions and policy interventions to ensure the sustainable development of CBEC ecosystems [26, 27]. Recent studies have examined various factors influencing CBEC, including marketing strategies, payment methods, consumer preferences, and government policies. Structural equation modeling and empirical analyses have been employed to understand the complex dynamics of CBEC transactions and customer experiences [28–30]. In response to the development needs of CBEC transaction models and trading platforms, research suggested the supply and demand gap analyses for the key competencies of CBEC personnel, strengthening the market orientation of CBEC, and absorbing and cultivating personalised e-commerce talent [30]. Research also examined that the definition and model development and the dilemmas encountered in CBEC [29]. Research has also focused on developing organizational strategies for CBEC and evaluating the development issues associated with CBEC and free trade zones. By examining the synergistic relationship between CBEC and free trade zones, scholars aim to identify potential issues and propose strategies for sustainable development [31]. The literature on CBEC and logistics performance provides valuable insights into the evolving dynamics of cross-border trade, the role of technological innovation, and the challenges and opportunities facing businesses and policymakers. By comprehensively reviewing recent studies, researchers can build on existing knowledge to advance theoretical frameworks, empirical models, and practical solutions for enhancing the efficiency and competitiveness of CBEC ecosystems.

### Effectiveness of cross-border logistics

Scholars have categorized CBEC and logistics research into four main aspects: logistics models, border e-logistics, cross-domain logistics, and collaborative development of CBEC logistics [5]. These categories encompass a wide range of studies exploring different facets of the interaction between CBEC and logistics, including operational models, regulatory frameworks, and collaborative strategies. CBEC is one of the popular topics in corporate logistics research [32]. Researchers have investigated various models and methods in CBEC logistics, including international express, postal parcel, and domestic express internationalization models [28, 33]. These studies provide insights into the diversity of logistics approaches adopted by CBEC enterprises and highlight the importance of selecting appropriate logistics models to optimize cross-border trade operations. Zhang (2018) classified the present state of cross-border logistics into three distinct categories including the international express model, the international postal parcel model, and the domestic express internationalisation model [33]. Subsequently, Yang et al. (2022) argued that cross-border logistics is one of the bottlenecks faced by CBEC’s enterprises when facing the CBEC rules of several countries [31]. Li’s (2014)’s study examined that the standard CBEC and logistics methods could be divided into three main types such as overseas warehouses, postal services, and international express delivery [28]. Li (2014) also stated that to further encourage the growth of CBEC’s logistics networks, nations and businesses should place a greater emphasis on creating foreign warehouses and enhancing logistics monitoring and top-level testing [34]. In respect to the formation of foreign warehouses, Zhang (2016) also opined that the construction of overseas warehouses is favourable to resolving the difficulties encountered in China’s CBEC’s logistics development [35].

Many CBEC enterprises rely on outsourcing as a business model to achieve synergistic development and aggregation effects in logistics operations [36]. Outsourcing enables enterprises to leverage the expertise and resources of logistics service providers, thereby enhancing efficiency and resource utilization across different logistical modes. The expansion of cross-border logistics in conjunction with CBEC is identified as a key trend for the future [37]. To adapt to this trend, businesses and governments should focus on integrating resources, optimizing logistics outsourcing models, and promoting collaboration between CBEC enterprises and regional logistics providers. To adjust to the growth of CBEC, businesses and governments should be committed. Challenges in cross-border logistics could hinder the growth of CBEC and lead to operational obstacles. To address these challenges, governments and enterprises should prioritize cooperation and coordinated development of cross-border logistics and e-commerce [38]. Exploring diverse models of cross-border logistics is essential for overcoming logistical bottlenecks and promoting the sustainable development of CBEC ecosystems. Collaboration between governments, enterprises, and logistics providers is crucial for addressing the evolving needs of CBEC logistics. Embracing innovation and exploring non-traditional models of logistics can help unlock new opportunities for cross-border trade and enhance the competitiveness of CBEC enterprises [36, 38].

### Synergistic development and cross-border e-commerce

Recent research highlights the significant impact of the supply chain on the synergistic development of CBEC and logistics. AI’s study emphasizes the role of the supply chain as a primary factor influencing the integration of resources and the achievement of strategic coordination patterns through vertical integration [39]. These findings also suggest further accelerated resource sharing of physical assets and skills between enterprises, which will ultimately result in the achievement of a strategic coordination pattern in vertical integration. Yu et al. propose optimization paths based on their research on CBEC and logistics cooperation, aiming to facilitate resource integration and the alignment of upstream and downstream elements in the supply chain [40]. These paths provide practical strategies for enhancing collaboration and improving supply chain efficiency. Scholars have qualitatively described and assessed the synergy between CBEC and logistics, emphasizing the importance of information and knowledge sharing for achieving cross-functional cooperation [41]. Studies by Mou analyze supply chain coordination interfaces and propose coordination mechanisms to enhance overall performance, considering centralized and decentralized decision-making models [42]. They studied two models in relation to the centralized decision-making, and decentralized decision-making, using collaborative mechanisms. For example, they used the principal-agent model to study supply chain coordination and proposed an incentive model for supply chain coordination to study the specific information-sharing assessment coefficients. Based on the results of qualitative studies, some studies have used quantitative analysis to validate the relationship between e-commerce and logistics further. Based on Granger causality tests and vector autoregressive models, logistics and e-commerce would have a mutual inhibitory effect in a short period. Some studies employ quantitative analysis to validate the relationship between e-commerce and logistics. Wang’s research reveals a cooperative pattern of interdependence between e-commerce and cross-border logistics enterprises, highlighting the dynamic nature of their interaction [6]. Dynamic evolutionary game models are utilized to study the competitive and cooperative dynamics between e-commerce and third-party logistics, shedding light on influencing factors and long-term stability [5]. Liu introduces a composite system synergy model to measure the degree of synergy between CBEC and logistics, providing a quantitative framework for assessing their interaction and evolution [5]. This model offers insights into the dynamic and cooperative nature of their relationship, facilitating a deeper understanding of their synergistic processes. Despite successful adoption of collaboration and coordination mechanisms in conventional supply chains, the coordinated growth of CBEC and logistics remains in its infancy. Understanding the consolidated factors of economic, social, technological, and knowledge diffusion is crucial for unlocking the full potential of their synergistic development [5]. Recent studies provide valuable insights into the complex dynamics of CBEC and logistics, emphasizing the importance of collaboration, resource integration, and supply chain coordination for achieving synergistic development. By synthesizing findings from qualitative and quantitative analyses, researchers can advance our understanding of the interplay between CBEC and logistics and inform strategies for enhancing their mutual benefits.

## Theoretical model and synergistic mechanisms

In the view of CBEC literature, synergy usually indicates a relational target that individual partners cannot attain alone; they must collaborate to attain it. Accordingly, the synergistic mechanisms necessitate that those involved businesses and logistics service providers collaborate on logistical activities and share a strategic objective when formulating organisational strategies to accomplish a mutually beneficial target [10]. Synergy is a dynamic process that necessitates adaptation and learning and produces an integrated solution. Specifically, it is the subsystems of a composite system that generate beneficial synergistic effects with the aid of a scientific and efficient coordination mechanism, which changes the composite system from a disordered installation to an ordered one, and vice versa [10].

In this context, the synergistic mechanism for CBEC is a shared strategic objective. It seeks to serve and assist consumers with goods and services of higher quality than the local e-commerce sector. For example, offline shopping attracts potential, and new customers to expand the CBEC through synergistic growth and achieve the expected benefits of CBEC and logistics. Both are internally and externally motivated to build CBEC-focused businesses through partnerships, with integration and synergy to deliver quality services to cross-border online customer.

### Dynamics of cross-border e-commerce

The synergistic mechanism in this study pertains to external and internal CBEC and logistical dynamics [43]. The external drivers refer primarily to the impact of the social environment on the CBEC and supply chain network, such as changes in market development, advances in information technology, and shifts in consumer attitudes. Technological advancement is one of the external forces promoting CBEC and logistics synergy, which enables cross-enterprise and cross-country synergy between CBEC and logistics [30]. Diversification and velocity of consumer demand have prompted efficient coordination between CBEC and logistical networks to fulfil consumer needs [29]. For this connection, internal motivation is measured as the desire to achieve synergy within the CBEC and supply chain network, including enterprises’ pursuit of high profits and the correlation between the nodes [44]. First, the specialised division of labour establishes the functional correlation between nodes, which enables enterprises to create competitive advantages through collaborative sharing. Second, businesses’ pursuit of profit maximisation is the fundamental driving force of supply chain synergy. The motivation for enterprises is to coordinate the supply chain, achieve business objectives, and reap synergistic benefits. By analysing market advantages and disadvantages, enterprises keep the core links at which they excel, outsource non-core connections or businesses that lack competitive advantages, and build synergistic ties with them to produce synergy and win-win outcomes.

### Modelling of the cross-border e-commerce

In this section, we focus on the modelling of the CBEC for the synergistic effect analysis and evaluate the usefulness and flexibility of CBEC to achieve competitive advantage. Firstly, the proposed model is to assess the direct manifestation of the synergetic effect between CBEC and logistical tasks, which is about the revenue of the enterprise and secondly, it then determines the synergistic operation of the subsystems in CBEC [29]. Specifically, it is to develop the understanding of synergistic behaviour, which contributes to greater profits gained than each subsystem operates independently.

The value-added benefit due to the synergistic behaviour represents the economic effect of the synergy between CBEC and logistical tasks. The mathematical expression for the synergistic effect, SE is derived as shown in Eq (1).

$$\left\{\begin{array}{c} SE=F\left(N\right)-\sum_{i=1}^{n}C\left(x_{i}\right)-\sum_{i=1}^{n}F(x_{i})\\ N=f\left(x_{1},x_{2},\ldots ,x_{n}\right) \end{array}\right.$$ (1)

where

*F*(*x*_*i*_) is the effect of each node enterprise, *x*_*i*_ when operating individually

*C*(*x*_*i*_) is the cost to be made by each nodal enterprise, *x*_*i*_ for the collaborative system

*F*(*N*) is the total effect of CBEC in a supply chain when systems work together. The total effects are divided into economic effects, knowledge and technology diffusion effects and social effects.

The pursuit of maximisation of mutual benefits is the activities are given behind the relationship between CBEC and logistics [4]. There are economic, and social effects and knowledge and technology diffusion that are summarised as follows.

- Economic effect: The effect on the economy considers the economies of scale and it is the synergy of CBEC and logistics for performance measurement. This synergy may be improved end-customer satisfaction and loyalty through the synergy in terms of benefit, information, capacity, and business process. As a result, the scale of online shopping is expanded, and the unit operating costs of CBEC and logistics are then reduced and then it could also result obvious economic benefits. The economy of scale effect is the integration and coordination of different businesses between CBEC and logistics. In addition, it enables enterprises to give full play to their existing resource advantages at lower costs, respond to demand more quickly, gradually build trust, reduce the costs and risks of cross-border online shopping transactions, and form a competitive advantage.
- Knowledge and technology diffusion: When one node adopts new knowledge, technology, and skills as part of the collaboration between CBEC and logistics, the other nodes that are related to it will learn by imitation through intensive communication. This makes it possible for the information and technical innovation to be dispersed, shared, and disseminated [3]. Because of this, nodes would be able to gain new knowledge and skills, which results in improved service capabilities. Subsequently, it lessens the cost of investment and the use of new information and technology.
- Social effect: The synergistic operation of CBEC and logistics have a favourable impact on the development of the relevant external economy and society, manifesting as a socio-economic effect and an environmental effect. The social effect generated by the positive synergy is a long-term strategic effect that is conducive to the sustainable development of cross-border e-commerce and logistics.

### Development of indicator selection

For developing the selection of indicators, the reasonable and effective index system is proposed to effectively measure the level of synergistic development between CBEC and logistics planning to ensure the accuracy of the measurement of the level of synergistic development of CBEC. As a result, this study makes use of research that was conducted based on the features of each sub-system, as well as the availability and dependability of the data. Table 1 presents the assessment indicators that were initially selected for the project. This study, obtaining the indicator system of Guangdong Province CBEC subsystem as X1, X2, X3, X4, X5\X6, X7, X8, X9 and X10, and the indicator system of cross-border logistics as Y1, Y2, Y3, Y4, Y5, Y6, Y7, Y8, Y9 and Y10. In developing our empirical model, we carefully considered a range of variables that we believe are pertinent to understanding the dynamic relationship between cross-border e-commerce (CBEC) and logistics co-development strategies for small and medium-sized enterprises (SMEs) in China. These variables were selected based on extensive literature review and expert consultation, aiming to capture the multifaceted nature of CBEC logistics and its implications for competitive advantage. X1 represents the total volume of cross-border e-commerce transactions in Guangdong Province, which serves as a fundamental measure of the scale of cross-border e-commerce activity in the region. X2, captures the total volume of import and export transactions in Guangdong Province, providing context for the overall trade activity in the region and its relationship with cross-border e-commerce. X3, measures the total volume of online shopping transactions conducted by residents of Guangdong Province, reflecting the consumer behavior and demand for e-commerce services in the region. X4 quantifies the annual growth rate of cross-border e-commerce transaction scale in Guangdong Province, indicating the rate of expansion or contraction of cross-border e-commerce activity over time. X5 assesses the contribution of cross-border e-commerce to the overall import and export activity in Guangdong Province, highlighting the significance of cross-border e-commerce in the region’s trade ecosystem. X6 represent contribution of cross-border e-commerce to import and export in Guangdong Province. X7 represents the consumption level of residents in Guangdong Province, providing insight into the purchasing power and consumer behavior of the local population. X8 denotes the total number of internet users in Guangdong Province, serving as an indicator of digital connectivity and potential market size for online services, including e-commerce. X9 represent Internet penetration rate in Guangdong Province. X10 reflects the size of the wholesale and retail workforce in Guangdong Province, which can influence the distribution and sales channels of cross-border e-commerce products.

**Table 1:** Initially identified indicator system.

*Sub-systems*	*Dimensions*	*Sequential parametric indicators*	*Variables*
*Cross-border e-commerce*	*Scale*	*Total cross-border e-commerce transactions in Guangdong Province*.	X_1_
		*Total import and export transactions in Guangdong Province (RMB billion)*.	X_2_
		*Total online shopping transactions in Guangdong Province (RMB billion)*	X_3_
	*Quality*	*Annual growth rate of cross-border e-commerce transaction scale in Guangdong Province (%)*.	X_4_
		*Penetration of cross-border e-commerce consumer goods in total retail sales of social consumer goods in Guangdong Province (%)*.	X_5_
		*Contribution of cross-border e-commerce to import and export in Guangdong Province (%)*.	X_6_
	*Potentials*	*Consumption level of Guangdong residents (RMB)*.	X_7_
		*Number of Internet users in Guangdong Province (million)*.	X_8_
		*Internet penetration rate in Guangdong Province (%)*.	X_9_
		*Employees in wholesale and retail trade in Guangdong Province (persons)*.	X_10_
*Cross-border logistics*	*Scale*	*Express volume in Guangdong Province (Million pieces)*.	Y_1_
		*Annual revenue of express delivery in Guangdong Province (RMB million)*.	Y_2_
		*Freight volume in Guangdong Province (Million tons)*.	Y_3_
		*Cargo turnover in Guangdong Province (Billion-ton kilometres)*.	Y_4_
		*Cargo throughput of major ports in Guangdong Province (million tons)*.	Y_5_
	*Infrastructure*	*Fixed investment in transport*, *storage and postal industry in Guangdong Province completed (RMB billion)*.	Y_6_
		*Number of berths at major ports in Guangdong Province*.	Y_7_
		*Length of major port terminals in Guangdong Province (meters)*.	Y_8_
		*Total railway mileage in Guangdong Province (km)*.	Y_9_
		*Total road mileage in Guangdong Province (km)*.	Y_10_

The variables selected encompass various dimensions of cross-border e-commerce, trade activity, consumer behavior, and economic indicators relevant to Guangdong Province. The inclusion of these variables aims to provide a comprehensive understanding of the factors influencing cross-border e-commerce and logistics co-development in the region.

In addition, Y1 represents the magnitude of courier volume in Guangdong Province, indicating the extent of logistics activity related to express delivery services within the region. Y2 measures the total income generated from express delivery services in Guangdong Province over a specific period, reflecting the economic value contributed by the express delivery sector to the regional economy. Y3 freight volume refers to the total volume of goods transported through various modes of transportation, such as air, sea, and land, within Guangdong Province. This variable provides insights into the overall demand for transportation services and logistics infrastructure in the region. Y4 captures the total turnover or sales volume of goods within Guangdong Province, indicating the level of commercial activity and demand for logistics services to facilitate the movement of goods within the region. Y5 measures the total volume of goods handled by major ports in Guangdong Province, reflecting the maritime trade activity and the importance of ports as key nodes in the regional logistics network. Y6 represents the total investment in infrastructure related to transport, storage, and postal services in Guangdong Province, including investments in roads, railways, warehouses, and postal facilities. It reflects the level of government and private sector investment in developing logistics infrastructure within the region. Y7 represent Number of berths at major ports in Guangdong Province. Y8 indicates the capacity of ports to accommodate vessels and handle cargo shipments. This variable reflects the infrastructure capacity of ports in Guangdong Province and their ability to support maritime trade activities. Y9 refers to the length of railway tracks or lines within Guangdong Province, indicating the extent of rail infrastructure and its role in facilitating freight transportation and logistics operations within the region. Y10 represents the length of road networks within Guangdong Province, including highways, expressways, and local roads. This variable reflects the extent of road infrastructure and its importance for land transportation and logistics connectivity within the region.

The selected variables encompass various aspects of cross-border logistics, including transportation infrastructure, freight volume, port operations, and logistics-related investments, providing a comprehensive framework for analyzing the dynamics of logistics co-development in Guangdong Province. The specific screened indicators are shown in Tables 1 and 2.

**Table 2:** Grey correlation matrix of CBEC and related indicators.

	X_1_	X_2_	X_3_	X_4_	X_5_	X_6_	X_7_	X_8_	X_9_	X_10_
Y_1_	0.7771	0.8910	0.9150	0.7956	0.8923	0.7204	0.8043	0.8964	0.6431	0.8989
Y_2_	0.8911	0.9361	0.9642	0.8308	0.9351	0.7199	0.9400	0.9427	0.6344	0.9452
Y_3_	0.9053	0.9777	0.9885	0.8558	0.9766	0.7135	0.9824	0.9857	0.6376	0.9880
Y_4_	0.8856	0.9891	0.9782	0.8504	0.9879	0.7193	0.9339	0.9928	0.6018	0.9984
Y_5_	0.8943	0.9695	0.9940	0.8527	0.9697	0.7034	0.9750	0.9777	0.6453	0.9779
Y_6_	0.8964	0.9765	0.9712	0.8472	0.9964	0.7189	0.9476	0.9946	0.6247	0.9922
Y_7_	0.8957	0.9167	0.9657	0.8323	0.9077	0.6968	0.9019	0.8986	0.6384	0.9163
Y_8_	0.8989	0.9780	0.9675	0.8440	0.8991	0.7089	0.9039	0.9906	0.6320	0.9182
Y_9_	0.7771	0.9893	0.9786	0.8495	0.9851	0.7193	0.9941	0.9920	0.6352	0.9572
Y_10_	0.8915	0.9984	0.9689	0.8448	0.9386	0.7035	0.9050	0.9420	0.6726	0.8796

#### Analysis and selection of indicators

As there are uncertainties in the development of the CBEC subsystem and cross-border logistics subsystem, the relevant sample data cannot fully reflect the meaning of each indicator. In order to evaluate it, the grey correlation analysis method is applied to screen the sequential covariate indicators to ensure a strong correlation between the subsystems [45]. The grey correlation analysis is used to determine the degree of correlation by the similarity of the geometric shapes between the curves of the indicators. The closer the curves are to each other, the greater the degree of correlation; conversely, the smaller the degree of correlation. The correlation formula is as shown in Eq (2)

$$\text{R}=\frac{1}{n}\sum_{j=1}^{n}\frac{\text{min}\;\text{min}\left|X_{i}\left(t\right)-Y_{i}\left(t\right)\right|+\lambda \;\text{max}\;\text{max}\left|X_{i}\left(t\right)-Y_{i}\left(t\right)\right|}{\left|X_{i}\left(t\right)-Y_{i}\left(t\right)\right|+\lambda \;\text{max}\;\text{max}\left|X_{i}\left(t\right)-Y_{i}\left(t\right)\right|}$$ (2)

*X*_*i*_(*t*) and *Y*_*i*_(*t*) represent the standardized values of CBEC and cross-border logistics sub-system indicators in the Guangdong Province at moment t, where λ is the discrimination coefficient and takes the value of 0.5. According to Eq (2), the grey correlation between CBEC and cross-border logistics sub-system indicators in the Guangdong province was derived using MATLAB software, as shown in Table 2.

As tabulated in Table 2, the correlation degree of each sequential parameter indicator of Guangdong province CBEC and cross-border logistics subsystems is relatively large, indicating that the correlation between the two is high. In this study, we adapt the correlation benchmark of 0.75 as the threshold value to screen the indicators of each subsystem, thus obtaining the indicator system of Guangdong Province CBEC subsystem as X_1_, X_2_, X_3_, X_4_, X_6_, X_7_, X_8_ and X_10_, and the indicator system of cross-border logistics as Y_1_, Y_2_, Y_3_, Y_4_, Y_5_, Y_6_, Y_9_ and Y_10_. The specific screened indicators are shown in Table 3.

**Table 3:** Filtered indicator system.

*Sub-systems*	*Dimensions*	*Sequential parametric indicators*	*Variables*
*Cross-border e-commerce*	*Scale*	*Total cross-border e-commerce transactions in Guangdong Province*.	X_1_
		*Total import and export transactions in Guangdong Province*.	X_2_
		*Total online shopping transactions in Guangdong Province*.	X_3_
	*Quality*	*Annual growth rate of cross-border e-commerce transaction scale in Guangdong Province*.	X_4_
		*Contribution of cross-border e-commerce to import and export in Guangdong Province*.	X_5_
	*Potentials*	*Consumption level of Guangdong residents*.	X_7_
		*Number of Internet users in Guangdong Province*.	X_8_
		*Wholesale and retail workers in Guangdong Province*.	X_10_
*Cross-border logistics*	*Scale*	*Courier volume in Guangdong Province*.	Y_1_
		*Annual income from express delivery in Guangdong Province*.	Y_2_
		*Freight volume in Guangdong Province*.	Y_3_
		*Turnover of goods in Guangdong Province*.	Y_4_
		*Cargo throughput of major ports in Guangdong Province*.	Y_5_
	*Infrastructure*	*Fixed investment in transport*, *storage and postal services in Guangdong Province*.	Y_6_
		*Number of berths at major ports in Guangdong Province*.	Y_8_
		*Total railway mileage in Guangdong Province*.	Y_9_
		*Total road mileage in Guangdong Province*.	Y_10_

#### Data description

Both the development of CBEC and the growth of cross-border logistics contribute to the performance improvement for SMEs. The development of CBEC can enhance the performance of cross-border logistics. Scholars have estimated that CBEC and cross-border logistics in Guangdong are at a low synergy stage [7]. An empirically model estimation was proposed to determine synergy between CBEC and cross-border logistics according to the following sequential process.

- Step 1: The contribution of the orderly covariate indicators to the cross-border e-commerce and cross-border logistics subsystems in Guangdong, as screened by the grey correlation analysis method.
- Step 2: Each indicator was assigned a weight by the Criteria Importance Through Intercriteria Correlation (CRITIC) method, and then the ordinality of the CBEC and cross-border logistics subsystems in the year of 2012 to 2018.
- Step 3: The synergy equations of the subsystems of CBEC and cross-border logistics are constructed based on the sequential covariate indicators and the weights assigned and the synergy degree of the composite system is measured and evaluated.

### Orderliness of cross-border -commerce: An empirically model estimation

This study assumes that the Guangdong CBEC system is sub-system *S*_1_ and the cross-border logistics system is sub-system S_2_, the combined system formed by the two is S, i.e., *S* = {*S*_1_, *S*_2_}. The synergistic development of *S*_1_ and *S*_2_ is fundamentally an increase of the orderliness of the system S. *e*_1_ = {*e*_*a*1_, *e*_*a*2_, …, *e*_*aj*_} is the sequential parameters used for the Guangdong CBEC system, *e*_2_ = {*e*_*b*1_, *e*_*b*2_, …, *e*_*bj*_} is the ordinal parameter of the Guangdong cross-border logistics system, where j denotes as the number of ordinal parameter indicators in the subsystem. *e*_*aj*_ and *e*_*bj*_ denote the sequential parameters of the CBEC system and cross-border logistics system in Guangdong Province, respectively, and their values range from *α*_*aj*_ ≤ e_*aj*_ ≤ *β*_*aj*_, *α*_*bj*_ ≤ e_*bj*_ ≤ *β*_*bj*_, (*β*_*ij*_ or *α*_*ij*_ is upper and lower limits of e_*ij*_ values). Based on the existing theories of the collaborators, the degree of contribution of the ordinal covariates to each subsystem is defined by constructing an efficacy function. It is then calculated as shown in Eq (3).

$$u_{i}\left(e_{ij}\right)=\left\{\begin{array}{ll} \frac{e_{ij}-\beta_{ij}}{\alpha_{ij}-\beta_{ij}}, & j=i,\;\ldots ,l\\ \frac{e_{ij}-\beta_{ij}}{\alpha_{ij}-\beta_{ij}}, & j=l+1,\ldots ,n \end{array}\right.$$ (3)

where *u*_*i*_(*e*_*ij*_) ∈ [0,1] and the larger its value, the greater the contribution of *e*_*ij*_ to the ordering of the system.

The contribution of the ordinal covariates in a subsystem to the total system order can be obtained by measuring the weighted average of the contribution of the ordinal covariates to the subsystem as derived in Eqs (4) and (5).

$$\begin{array}{ll} u_{i}\left(e_{i}\right)=\sum_{j=1}^{n}w_{i}u_{i}\left(e_{ij}\right) & i=1,2\ldots \end{array}$$ (4)

$$\begin{array}{ll} w_{i}\geq 0, & \sum_{j=1}^{n}w_{i}=1 \end{array}$$ (5)

where *w*_*i*_ is the weight of each ordinal covariate indicator and *u*_*i*_(*e*_*i*_) is the ordinality of subsystem S_*i*_, taking the values [0, 1], reflecting the contribution of the cross-border e-commerce logistics composite system S. The size measure of *w*_*i*_ can be assigned to it using the CRITIC assignment method. The sample studied in this paper is a time series sample, and the CRITIC method considers the influence of the size of the indicator transformation on the weights, which is more objective and comprehensive compared to the entropy weighting method and the outlier assignment method. Its metaethical expression is derived as Eqs (6) and (7).

$$\begin{array}{ll} C_{ij}=\sigma_{j}\sum_{j=1}^{n}\left(1-\rho_{ij}\right) & i=1,2,\ldots ,k;j=1,2,\ldots ,n \end{array}$$ (6)

$$\begin{array}{ll} w_{i}=\frac{C_{ij}}{\sum_{j=1}^{n}C_{ij}} & i=1,2,\ldots ,k;j=1,2,\ldots ,n \end{array}$$ (7)

where *C*_*ij*_ represents the degree of influence of the j-th indicator on the whole system of evaluation indicators, and *σ*_*j*_ is the standard deviation of the jth evaluation indicator, i.e., the number of correlation coefficients derived from the grey correlation matrix above. the larger the *C*_*ij*_ the greater the relative importance of the indicator.

### An empirically synergy model of cross-border e-commerce

The relationship between cross-border e-commerce and cross-border logistics is in a dynamic process of change, changing over time, the synergy between cross-border e-commerce and cross-border logistics in Guangdong must be measured dynamically. Assume that at a particular stage *t*_0_, the orderliness of the Guangdong cross-border e commerce system and the cross-border logistics system is $u_{i}^{t_{o}}\left(e_{i}\right)$, and when the system develops to time *t*_1_, the orderliness of both subsystems *S*_*i*_ is $u_{i}^{t_{1}}\left(e_{i}\right)。$. It is calculated as.

$$\text{U}=\sqrt[{n}]{\prod_{1}^{n}|u_{i}^{t_{1}}(e_{i})-u_{i}^{t_{o}}\left(e_{i}\right)|}$$ (8)

where the synergy size is denoted by U, which takes values in the range [0, 1]. When U = 1, the system is extremely synergistic; when U = 0, the system is extremely non-synergistic. If $u_{i}^{t_{1}}\left(e_{i}\right)>u_{i}^{t_{o}}\left(e_{i}\right)$, then the whole composite system is in positive synergy within [*t*_0_, *t*_1_] and both have positive synergy effect.

## Results for an empirically model estimation

Based on the filtered ordinal parameters, the weights of the ordinal parameter indicators are calculated. According to the formula and the obtained ordinal parameter weights, the orderliness of the subsystem is calculated. The calculation results are shown in Table 4.

**Table 4:** Weights of sequential covariate indicators for CBEC and cross-border logistics systems.

*Sub-systems*	*Dimensions*	*Weights*	*Sequential parametric indicators*	*Weights*
*Cross-border e-commerce*	*Scale*	*0*.*4614*	*Total cross-border e-commerce transactions in Guangdong Province*.	*0*.*3658*
			*Total import and export transactions in Guangdong Province*.	*0*.*0587*
			*Total online shopping transactions in Guangdong Province*.	*0*.*0369*
	*Quality*	*0*.*2536*	*Annual growth rate of cross-border e-commerce transaction scale in Guangdong Province*.	*0*.*1570*
			*Contribution of cross-border e-commerce to import and export in Guangdong Province*.	*0*.*0966*
	*Potentials*	*0*.*2850*	*Consumption level of Guangdong residents*.	*0*.*1133*
			*Number of Internet users in Guangdong Province*.	*0*.*0576*
			*Wholesale and retail workers in Guangdong Province*.	*0*.*1141*
*Cross-border logistics*	*Scale*	*0*.*5340*	*Courier volume in Guangdong Province*.	*0*.*2101*
			*Annual income from express delivery in Guangdong Province*.	*0*.*1009*
			*Freight volume in Guangdong Province*.	*0*.*0648*
			*Turnover of goods in Guangdong Province*.	*0*.*0833*
			*Cargo throughput of major ports in Guangdong Province*.	*0*.*0748*
	*Infrastructure*	0.4660	*Fixed investment in transport*, *storage and postal ser*.	*0*.*0787*
			*Number of berths at major ports in Guangdong Province*.	*0*.*1150*
			*Total railway mileage in Guangdong Province*.	*0*.*1500*
			*Total road mileage in Guangdong Province*.	*0*.*1223*

As seen in Table 4, the scale of CBEC transactions, the annual growth rate of cross-border e-commerce transactions, and the number of Internet users in Guangdong significantly influence the orderliness of the cross-border e-commerce system.

In Guangdong’s cross-border logistics system, there is not much difference in the influence of each ordinal parameter indicator on the degree of orderliness, and the main ordinal parameter indicators with slightly greater influence are the total amount of express delivery and income of the express delivery industry in Guangdong. The number of berths in important ports, and railway mileage and kilometre mileage in Guangdong. In terms of dimensions, the scale has more than half the impact on the orderliness of cross-border e-commerce and cross-border logistics systems and increasing the indicators of the scale dimension can improve system synergy quickly. Therefore, construction can focus on expanding the scale of CBEC transactions and increasing infrastructure construction to promote the better-coordinated development of cross-border e-commerce and logistics.

The finding indicated that scale of CBEC transactions is likely a crucial factor as larger transaction volumes can introduce complexities in processing, logistics, and customs clearance. Higher transaction volumes may require more robust organizational structures to maintain orderliness. It implies that as the scale of transactions increases, there might be a need for more sophisticated systems, streamlined processes, and advanced technologies to manage the complexity efficiently. Secondly, the annual growth rate is an indicator of the dynamic nature of CBEC. A rapidly growing market may face challenges related to adapting existing systems to accommodate increasing demand. This can impact the orderliness of the system. A high growth rate might necessitate proactive measures, such as continuous system upgrades, scalability planning, and workforce training to maintain orderliness. Third, the number of Internet users reflects the user base engaging in online transactions. A higher number of users can lead to increased diversity in preferences, demands, and transaction types, requiring a more adaptable and organized system.

Moreover, in the logistics system, the total amount of express delivery is a key indicator. Its influence on orderliness might be more evenly distributed as express delivery is a critical component in the logistics chain, and variations in its scale can impact overall efficiency. A balanced focus on managing and optimizing express delivery services, including route planning, package tracking, and delivery speed, contributes to maintaining orderliness across the logistics system.

The findings suggest that the CBEC system is influenced by specific factors related to transaction scale, growth rate, and user base, highlighting the need for adaptability and sophistication. In contrast, the logistics system appears to be influenced more uniformly by indicators related to express delivery, emphasizing the overarching importance of an efficient logistics chain in maintaining orderliness. The differences in influencing factors between the cross-border e-commerce and logistics systems in Guangdong suggest the need for tailored management strategies to address the unique challenges and dynamics in each subsystem. This nuanced understanding can guide policymakers, businesses, and stakeholders in optimizing the efficiency and orderliness of cross-border operations in the region.

### Orderliness calculation for cross-border e-commerce

**Fig 1:**
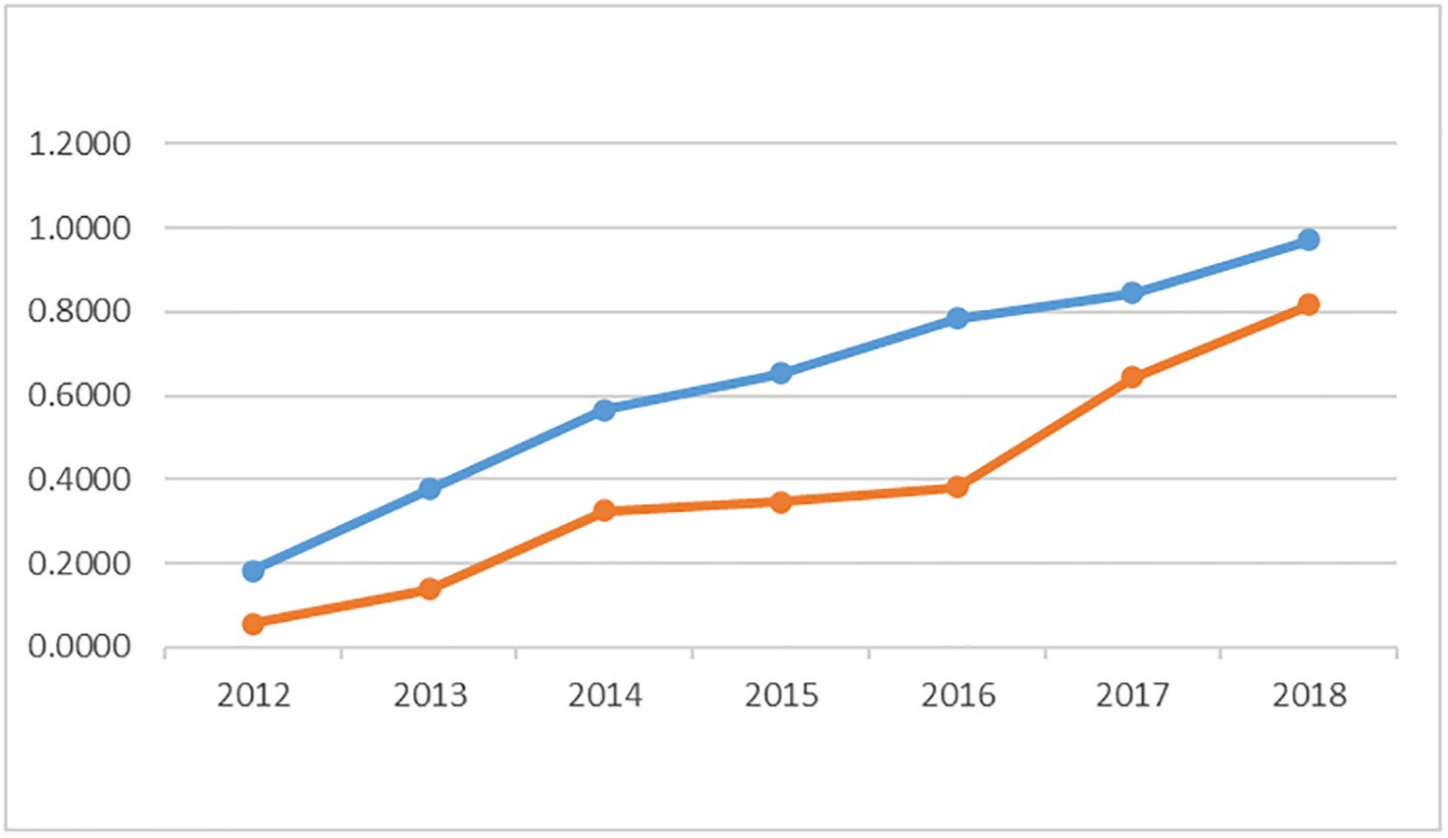
Discounted degree of orderliness of cross-border e-commerce and cross-border logistics subsystems.

The results of the orderliness calculations for the CBEC and cross-border logistics subsystems are shown in Table 5. Fig 1 shows the discounted degree of orderliness of CBEC and cross-border logistics subsystems.

**Table 5:** Orderliness of cross-border e-commerce and cross-border logistics subsystems.

Year	2012	2013	2014	2015	2016	2017	2018
Cross-border Logistics	0.1834	0.3773	0.5658	0.6537	0.7839	0.8447	0.9715
Cross-border e-commerce	0.0559	0.1376	0.3254	0.3475	0.3819	0.6436	0.8168

The obtained result shows that the orderliness of the CBEC subsystem and the cross-border logistics subsystem in Guangdong are on the rise, with the orderliness of the cross-border logistics subsystem being significantly higher than that of the CBEC subsystem. Moreover, the rise of the CBEC subsystem is flatter than that of the cross-border logistics system. In particular, the increase was relatively small during 2014–2016, but 2017 ushered in a significant increase of up to 88.53%. The growth of Internet users and the scale of cross-border e-commerce exports contributed to a larger increase in orderliness. Thus, we conclude that the popularity of the Internet and the expansion of export trade have driven the development of CBEC in the Chinese province.

The result indicating that the orderliness of the cross-border logistics subsystem in Guangdong is significantly higher than that of the cross-border e-commerce (CBEC) subsystem suggests a noteworthy trend. Guangdong have made substantial investments in physical infrastructure such as transportation networks, customs facilities, and logistics hubs. This enhanced infrastructure could contribute to the higher orderliness in the logistics subsystem. Secondly, stringent regulations and complex compliance processes in the CBEC subsystem may contribute to a lower orderliness score. Cross-border logistics may benefit from more streamlined regulatory processes. Third, the cross-border logistics subsystem may have integrated advanced technologies, such as tracking systems, route optimization, or warehouse automation, leading to improved orderliness.

### Synergy calculation for cross-border e-commerce

The results of the calculation of the synergy between the CBEC and cross-border logistics sub-systems based on the Eq (8) are shown in Table 6.

**Table 6:** Synergy between CBEC and cross-border logistics subsystems.

Year	2012	2013	2014	2015	2016	2017	2018
Cross-border e-commerce logistics system synergy	-	0.1259	0.1881	0.0441	0.0669	0.1261	0.1482

By comparing the obtained results in the year of 2013, the synergy has significantly increased in 2018. The overall synergy between CBEC and logistics in Guangdong shows not a linear increasing trend like the respective orderliness, but rather a wave-like spiral. This contrasts with the overall orderliness. In addition, the synergy between CBEC and cross-border logistics in Guangdong is still at a relatively low level, with a maximum of 0.1482, and there is still much room for improvement.

## Discussion

The findings underscore the critical importance of well-developed infrastructure, aligning with existing literature that emphasizes the significance of robust physical infrastructure for efficient cross-border operations [18, 19]. While previous research may acknowledge the importance of both cross-border e-commerce and logistics, our observation of disparities in orderliness levels between these subsystems in Guangdong presents a unique insight. Existing literature may not explicitly detail such variations in subsystem performance. Technological advancements likely facilitated improved communication, real-time tracking, and coordination between CBEC and logistics, while policy changes favoring cross-border operations may have further enhanced synergy.

The wave-like spiral pattern in synergy levels suggests periodic fluctuations influenced by external factors such as global economic conditions, geopolitical changes, or technological disruptions impacting the cross-border business environment. However, the relatively low synergy levels indicate ample room for improvement in integrating and collaborating between CBEC and logistics in Guangdong. Persistent challenges such as regulatory complexities, information asymmetry, or insufficient technological integration may hinder optimal synergy levels.

Guangdong, as one of the pioneering provinces for cross-border e-commerce, has become a hub for CBEC sellers, significantly enhancing its position in foreign trade. However, the rapid development of CBEC in Guangdong has exposed challenges in its supply chain system. Particularly, the lack of synergy between CBEC and cross-border logistics poses a significant obstacle, with empirical calculations revealing relatively low synergy levels, hindering overall supply chain efficiency and impeding the broader development of CBEC in Guangdong.

Addressing these challenges requires concerted efforts in three main areas: regulatory frameworks, technological integration, and information sharing. Policymakers and practitioners must prioritize initiatives to streamline regulations, enhance technological infrastructure, and promote greater collaboration and information exchange between CBEC and logistics stakeholders. By doing so, Guangdong can unlock the full potential of its CBEC ecosystem and maintain its competitive edge in the global market.

### No synchronized information sharing of cross-border e-commerce

Cross-border e-commerce and cross-border logistics have not built a suitable mutual trust mechanism. The inability to form a situation of information sharing has restricted the synergy and made the development more difficult. For example, in the actual trading activities of cross-border e-commerce and cross-border logistics enterprises lack trust. In the cooperation, both sides are worried about the leakage of their customer information and commercial secrets, resulting in both sides being unwilling to share information and data, which makes it more difficult to collaborate at the business level.

From the perspective of CBEC. Firstly, due to the lack of reliable information, enterprises cannot effectively assess the overall capacity of cross-border logistics enterprises. This makes difficult for enterprises to make accurate planning and timely adjustments. Indeed, may result in reducing the logistics experience of consumers. Secondly, enterprises cannot easily track the information of customers and the storage of goods in cross-border logistics promptly, may result in receiving feedback. Third, enterprises cannot track customer information and the storage of commodity goods in the cross-border logistics chain promptly, they are slow to accept feedback information, which makes cross-border e-commerce enterprises unable to control the delivery time within a reasonable range. This will affect the efficiency of delivery services and reduce consumers’ trust and satisfaction with cross-border e-commerce enterprises.

From the perspective of cross-border logistics enterprises. As they do not have access to information related to enterprises and cannot estimate the transaction scale of cross-border e-commerce enterprises according to development degree. This is not conducive to the development of the layout of cross-border logistics enterprises and affects the construction of the logistics service system of cross-border logistics enterprises. The development of cross-border logistics requires the support of the real economy and ample time to solve a series of problems, such as site selection, the establishment of warehouses, and the transportation of logistics equipment. The inability to know the information of cross-border e-commerce development in advance will not allow cross-border logistics resources to be reasonably allocated, and logistics resources will not be effectively utilized. Thus, leading to a waste of resources. For example, as a commercial secret, cross-border e-commerce companies are reluctant to share internal information such as order volumes and the amount and geographical scope of pre-sale activities with cross-border logistics enterprises. This leads to cross-border logistics being unable to grasp the trends of cross-border e-commerce enterprises in advance accurately. It brings difficult to meet demand for logistics services when enterprises are doing promotional activities. Indeed, resulting in many goods being burst, lost, or damaged. In the off-season, cross-border logistics enterprises have a surplus supply of logistics services, and empty warehouses appear.

The difficulty of not being able to synchronize and share information between cross-border e-commerce and cross-border logistics enterprises may be gradually reduced with the development of high technology such as big data, cloud computing, and Internet of Things technology. Technology development only alleviates the problem but does not fundamentally solve the problems. The root cause of the inability to share information synchronously between enterprises is the lack of mutual trust between enterprises. Therefore, establishing a suitable mutual trust mechanism between cross-border e-commerce and cross-border logistics enterprises is an effective measure to ensure synergistic developments.

### Lack of reverse logistics synergy of the cross-border e-commerce

Reverse logistics is an integral part of cross-border e-commerce services. Once a consumer returns product, the issue of reverse logistics arises, i.e., the goods need to flow back from the consumer to the cross-border e-commerce party in the opposite direction [6]. Compared to domestic e-commerce, the supply chain of the cross-border e-commerce industry is longer, involving more trading entities and more complex transaction process. As a result, it will take longer to resolve consumer returns and incur higher transaction costs.

As people’s living standards improve and consumers become more aware of rights and interests, consumers are demanding a higher level of quality and personalized experience from products [37]. However, according to the current development of cross-border e-commerce in Guangdong, cross-border e-commerce enterprises have not yet established good brand awareness. As a result, the products in the cross-border e-commerce market do not stand up to scrutiny regarding quality and personalization, and product homogenization is serious. This is contrary to the needs of the consumer market and has led to an increase in the probability and rate of returns, resulting in a growing demand for reverse logistics in the cross-border e-commerce market. At the same time, cross-border e-commerce enterprises hope to solve the problem of returns and exchanges at low cost and to achieve reverse logistics at low prices. For small items, the cost of reverse logistics may exceed the goods’ value. This makes cross-border logistics providers reluctant to develop reverse logistics for benefit.

In this paradox, cross-border e-commerce companies have not established long-term brand awareness. Enterprises focus more on short-term interests. After-sales service have not been given enough attention. This leads to less investment in the construction of reverse logistics services and does not promote the development of reverse logistics well. In turn, the development of reverse logistics is slow, while the development of cross-border e-commerce in Guangdong is rapid, and the level of synergy is low. Regarding the external environment for resolving conflicts, there are currently having no corresponding laws in China to regulate the level of after-sales service of cross-border e-commerce. An excellent after-sales service guarantee system has not yet been formed. Therefore, the problem of reverse logistics in the cross-border e-commerce supply chain cannot be changed in a short period.

### Incompatible levels of development of cross-border e-commerce

The development of the Internet has brought many technical advantages to cross-border e-commerce [9]. With the gradual application of information technology, network technology, and big data. Cross-border e-commerce in Guangdong is developing rapidly, and its demand for cross-border logistics parties is growing, which also puts higher requirements on the quality and service level of cross-border logistics [8]. Meanwhile, due to the innovation and breakthrough of Internet information technology, the concept of consumer demand has also changed accordingly, showing the characteristics of decentralization, diversification, and personalization. Cross-border e-commerce in the process of adapting to these characteristics has brought challenges to the operation of cross-border logistics [42]. However, these internet technologies cannot be connected to cross-border logistics in a short period, making it difficult for cross-border logistics to cater to the comprehensive development of cross-border e-commerce. Moreover, traditional cross-border logistics can hardly meet the needs of cross-border e-commerce for logistics, which restricts the development of cross-border e-commerce.

Logistics infrastructure has increased in all countries in recent years, with China bearing the brunt [46, 47]. Guangdong is accelerating logistics infrastructure construction and is committed to building an international logistics centre [7]. The development level of cross-border logistics in Guangdong is still at a low level. Most of the logistics enterprises in the province being small in scale, low in specialization, and providing a single form of logistics service, make it challenging to meet the comprehensive supply chain logistics solutions required by e-commerce [6]. Unfortunately, few large-scale logistics service enterprises in the Guangdong could provide more complete logistics services, such as SF, China Postal Express, and FedEx [8]. As a result, it is difficult for cross-border logistics to achieve seamless coordination and provide humanized logistics services for cross-border e-commerce in Guangdong. Consumer groups for cross-border e-commerce in Guangdong will pay more and more attention to the pursuit of personalized products and services, and the requirements of the seller’s market will be more stringent. This makes it more difficult for cross-border logistics to adapt to the development needs of cross-border e-commerce in Guangdong. When the level of cross-border logistics development is not coordinated with cross-border e-commerce, it will result in a lack of synergy.

## Theoretical contributions

The theoretical contribution provided by this manuscript is mainly reflected in the fact that it enriches its theoretical framework by incorporating supply chain management theories, such as the Resource-Based View (RBV). The RBV emphasises the role of internal resources and capabilities in achieving sustainable competitive advantage. Applied to cross-border e-commerce and logistics, this theory provides a perspective for analysing how the alignment and utilisation of resources within a supply chain can promote synergistic development. Our study goes beyond the traditional international trade model to incorporate supply chain management theory. By doing so, this paper provides a more comprehensive understanding of the synergistic relationship between cross-border e-commerce and logistics. This broadened theoretical perspective enriches the discussion around cross-border business strategies and contributes to theoretical advances in the field.

This study provides novel insights. Our research analyses highlight the dynamic interplay of resources and capabilities in the cross-border e-commerce and logistics domains. By identifying key resources and examining how they can be utilised to achieve synergies, the research provides novel insights into the strategic management of cross-border business. This nuanced understanding reveals the drivers of competitive advantage for Chinese SMEs. The research provides insights into the sustainability of competitive advantage resulting from the synergistic development of cross-border e-commerce and logistics. By integrating resources and capabilities across the supply chain, SMEs can build resilience, adaptability, and innovation. This focus on sustainability emphasises the long-term impact of synergistic development on firms’ success in the changing cross-border trade environment.

## Practical contributions and recommendations

Combining the practical problems of the synergistic development of cross-border e-commerce and cross-border logistics in Guangdong, this paper gives the following suggestions for enhancing the synergy.

### Building a mutual trust mechanism between cross-border e-commerce

To enhance the synergy between cross-border e-commerce and logistics in Guangdong, it is imperative to prioritize the establishment of a robust mutual trust mechanism. This mechanism should be designed to address key challenges and promote sustainable development in the cross-border e-commerce supply chain. Actionable recommendations for policymakers and practitioners as follow.

First, implement measures to facilitate information sharing between cross-border e-commerce enterprises and logistics providers. This will effectively control logistics and information costs for e-commerce businesses, leading to lower sales costs and increased profits. Additionally, it will enhance the logistics experience for customers and contribute to building a strong brand image for cross-border e-commerce companies.

Second, foster long-term strategic partnerships between cross-border e-commerce and logistics enterprises. Encourage information sharing to support the strategic regional layout of logistics operations based on e-commerce business strategies. This collaboration will optimize resource allocation, reduce risks, and drive innovation in cross-border logistics, ensuring its lasting vitality and sustainable development.

Third, develop a cooperation system between cross-border e-commerce and logistics entities, focusing on building a mutual trust mechanism. This should include provisions for compensating breaches of contract and the involvement of impartial third parties to facilitate contract resolutions. Strengthening trust between stakeholders will promote smoother transactions and foster a stable cross-border e-commerce supply chain.

Then, advocate for the establishment of local policies in Guangdong that safeguard cooperation between cross-border e-commerce and logistics firms. Enact regulations to facilitate legal and reasonable resolutions in the event of disputes between these entities. Clear legal frameworks will provide certainty and confidence for businesses operating in the cross-border e-commerce ecosystem.

Last, encourage regular communication and collaboration between policymakers, practitioners, and industry stakeholders to address emerging challenges and seize opportunities in the cross-border e-commerce landscape. Facilitate knowledge sharing and best practices exchange to drive continuous improvement in supply chain efficiency and effectiveness.

By implementing these recommendations, policymakers and practitioners can lay the foundation for a stronger and more resilient cross-border e-commerce supply chain in Guangdong. Building trust and fostering collaboration between e-commerce and logistics players will be pivotal in realizing the full potential of cross-border trade and ensuring sustainable growth in the region.

### Building an integrated network platform to promote reverse logistics synergies

To foster greater synergy between cross-border e-commerce and logistics in Guangdong, the establishment of an integrated network platform is paramount. The platform will optimize resource utilization and streamline cross-border logistics processes, thereby addressing reverse logistics challenges and enhancing consumer satisfaction throughout the shopping experience. Actionable recommendations for policymakers and practitioners as follow.

First, cross-border logistics enterprises should prioritize the optimization and refinement of logistics processes. This includes streamlining key steps such as order picking, packaging, labeling, customs clearance, delivery, and after-sales service. By minimizing delays and inefficiencies, the entire transaction process can be expedited, leading to improved consumer satisfaction.

Second, Guangdong government should take the lead in establishing integrated and unified platforms for cross-border logistics. Currently, numerous fragmented logistics companies operate independently, leading to inefficiencies and lack of interoperability. By creating a centralized platform and one-stop service system, information exchange and resource integration can be facilitated, enhancing efficiency and traceability.

Third, construction of an integrated network for cross-border logistics will significantly reduce operating costs for logistics enterprises. This reduction in costs creates an opportunity to address the challenge of reverse logistics between cross-border e-commerce and logistics. Collaboratively, cross-border logistics enterprises can establish logistics centers dedicated to handling returned goods, optimizing reverse logistics management and promoting synergistic development.

By implementing these recommendations, policymakers and practitioners can foster a more integrated and efficient cross-border e-commerce ecosystem in Guangdong. This approach will not only enhance operational efficiency and reduce costs but also contribute to the sustainable growth of cross-border trade and logistics in the region.

### Focus on composite talent training

Talent plays a pivotal role in driving the synergistic development of cross-border e-commerce and logistics. To address the need for composite talents in these fields, the Guangdong government can implement a dual strategy focusing on training and talent attraction. Actionable recommendations for policymakers and practitioners as follow.

First, leverage the educational resources of universities to create a high-level practical training platform. The government can lead the establishment of a comprehensive talent training model for the cross-border e-commerce supply chain, with enterprises as the primary stakeholders and colleges providing support. This model should emphasize the coordinated training of both theoretical knowledge and practical skills, fostering a talent pipeline with diverse expertise levels. Encourage students to acquire skills certificates in cross-border e-commerce and relevant qualifications in cross-border logistics management to enhance their employability.

Second, develop a comprehensive talent introduction mechanism to attract high-level professionals in the cross-border e-commerce industry to Guangdong. Encourage cross-border e-commerce and logistics enterprises to offer attractive training benefits to their employees and invest in enhancing the overall workforce quality through internal training programs. By nurturing and attracting talent, Guangdong can fortify its cross-border e-commerce and logistics sectors, driving synergistic development.

By implementing these recommendations, policymakers and practitioners can cultivate a skilled workforce equipped to drive innovation and efficiency in cross-border trade and logistics. This proactive approach to talent development will contribute to the sustained growth and competitiveness of Guangdong’s cross-border e-commerce ecosystem.

## Conclusion

The interdependence between Cross-Border E-commerce (CBEC) and cross-border logistics is irrefutable, each shaping the trajectory of the other’s evolution. This symbiotic relationship is underscored by CBEC’s influential position, amplified by the advantageous stance of e-merchants. However, realizing the full potential of this relationship necessitates a collaborative framework founded on mutual trust. While CBEC bears a lower cost for unilateral defaults, establishing a robust collaboration is imperative for both CBEC and cross-border logistics enterprises.

In the pursuit of a harmonious evolution, the imperative is to transcend unilateral approaches and establish mechanisms that foster mutual trust. Governments and industry stakeholders play pivotal roles in this Endeavor. Collaborative frameworks need to be devised, offering incentives and policies that encourage joint initiatives between CBEC and logistics entities. This collaborative ethos is central to enhancing the overall efficiency of cross-border operations, creating an ecosystem where both entities thrive in tandem.

One of the cornerstones of this collaborative framework is the establishment of enhanced mechanisms for mutual trust. Transparent communication, standardized processes, and the incorporation of cutting-edge technologies like blockchain can alleviate uncertainties inherent in cross-border transactions. Such mechanisms not only streamline operations but also contribute to building a foundation of trust between CBEC and logistics partners, paving the way for sustained collaboration.

In the policy realm, it is imperative for governments to prioritize continued investment in infrastructure, particularly in logistics and customs facilities. This commitment is essential to facilitate smoother cross-border transactions, reducing bottlenecks that might impede the fluidity of CBEC and logistics operations. Investment in infrastructure acts as a catalyst, propelling the collaborative framework towards greater effectiveness and efficiency.

However, it is crucial to acknowledge the limitations inherent in this study. Contextual factors that may affect the generalisability of the study results, such as the unique socio-economic environment of Guangdong Province and the specific characteristics of SMEs. The regional, cultural and economic context of China, while illustrative, may not encapsulate the complexities of other regions. We recognise the inherent diversity and complexity of CBEC and logistics environments, which can vary significantly across regions and industries. While our study provides valuable insights into the synergistic development of cross-border e-commerce and logistics strategies for Chinese SMEs, the dynamics of cross-border e-commerce and logistics can be affected by a variety of factors, including regulatory frameworks, infrastructure, market dynamics, and cultural differences, which can vary considerably across different environments.

Future research could take into account the diversity and complexity of CBEC and logistics environments in different regions and industries. Context-specific research is essential to fully understand the drivers and challenges of CBEC adoption and the effectiveness of logistics co-development strategies in different environments. To further enrich our understanding, future research efforts should explore the impact of evolving international trade regulations on the CBEC-cross-border logistics relationship. Such research can identify the challenges and opportunities presented by the dynamic policy environment and provide insights for the development of future strategies. In addition, cross-industry comparative analyses can provide a broader perspective on the interaction between CBEC and cross-border logistics, revealing differences and identifying best practices.

In conclusion, while this study emphasizes the symbiotic relationship between CBEC and cross-border logistics, the path to sustained growth lies in collaborative frameworks and mutual trust mechanisms. Policymakers, industry stakeholders, and scholars alike should heed these insights and recommendations to cultivate an environment conducive to the continued evolution of cross-border operations. In nurturing synergy, we will pave the way for a future where CBEC and cross-border logistics not only coexist but flourish together.

## Supporting information

S1 Dataset (XLS)
